# Long-Term Outcomes of Prostate-Specific Membrane Antigen–PET Imaging of Recurrent Prostate Cancer

**DOI:** 10.1001/jamanetworkopen.2024.40591

**Published:** 2024-10-23

**Authors:** Natalia Kunst, Jessica B. Long, Sarah Westvold, Preston C. Sprenkle, Isaac Y. Kim, Lawrence Saperstein, Maximilian Rabil, Umar Ghaffar, R. Jeffrey Karnes, Xiaomei Ma, Cary P. Gross, Shi-Yi Wang, Michael S. Leapman

**Affiliations:** 1 Centre for Health Economics, University of York, York, United Kingdom; 2 Cancer Outcomes, Public Policy, and Effectiveness Research (COPPER) Center, Yale School of Medicine, New Haven, Connecticut; 3 Department of Urology, Yale School of Medicine, New Haven, Connecticut; 4 Department of Radiology and Biomedical Imaging, Yale School of Medicine, New Haven, Connecticut; 5 Department of Urology, Mayo Clinic, Rochester, Minnesota; 6 Department of Chronic Disease Epidemiology, Yale School of Public Health, New Haven, Connecticut; 7 Department of Medicine, Yale School of Medicine, New Haven, Connecticut

## Abstract

**Importance:**

Although prostate-specific membrane antigen positron emission tomography (PSMA-PET) has shown improved sensitivity and specificity compared with conventional imaging for the detection of biochemical recurrent (BCR) prostate cancer, the long-term outcomes of a widespread shift in imaging are unknown.

**Objective:**

To estimate long-term outcomes of integrating PSMA-PET into the staging pathway for recurrent prostate cancer.

**Design, Setting, and Participants:**

This decision analytic modeling study simulated outcomes for patients with BCR following initial definitive local therapy. Inputs used were from the literature and a retrospective cohort study conducted at 2 institutions. The base case analysis assumed modest benefits of earlier detection and treatment, and scenario analyses considered prostate-specific antigen (PSA) level at imaging and different outcomes of earlier vs delayed treatment. The analysis was performed between April 1, 2023, and May 1, 2024.

**Exposures:**

(1) Immediate PSMA-PET imaging, (2) conventional imaging (computed tomography and bone scan [CTBS]) followed by PSMA-PET if CTBS findings were negative or equivocal, and (3) CTBS alone.

**Main Outcomes and Measures:**

The main outcomes were detection of metastases, deaths from prostate cancer, and life-years and quality-adjusted life-years (QALYs) gained.

**Results:**

The model estimated that per 1000 simulated patients with BCR (assumed median age, 66 years), PSMA-PET is expected to diagnose 611 (95% uncertainty interval [UI], 565-656) patients with metastasis compared with 630 (95% UI, 586-675) patients diagnosed using CTBS followed by PSMA-PET and 297 (95% UI, 202-410) patients diagnosed using CTBS alone. Moreover, the estimated number of prostate cancer deaths was 512 (95% UI, 472-552 deaths) with PSMA-PET, 520 (95% UI, 480-559 deaths) with CTBS followed by PSMA-PET, and 587 (95% UI, 538-632 deaths) with CTBS alone. Imaging with PSMA-PET yielded the highest number of QALYs, which were 824 (95% UI, 698-885) higher than CTBS. These results differed by PSA level at the time of testing, with the highest incremental life-years and QALYs and lowest number of deaths from prostate cancer among patients with PSA levels of at least 5.0 ng/mL. Finally, the estimates were sensitive to the expected benefit of initiating therapy for recurrent prostate cancer earlier in the disease course.

**Conclusions and Relevance:**

The results of this decision-analytic model suggest that upfront PSMA-PET imaging for the evaluation of BCR is expected to be associated with reduced cancer mortality and gains in life-years and QALYs compared with the conventional imaging strategy, assuming modest benefits of earlier detection and treatment.

## Key Points

**Question:**

What are the long-term outcomes of prostate-specific membrane antigen positron emission tomography (PSMA-PET) vs conventional imaging strategies in patients with recurrent prostate cancer?

**Findings:**

This decision-analytic modeling study of simulated patients with biochemical recurrent prostate cancer estimated that upfront PSMA-PET may lead to 75 fewer deaths from prostate cancer, 988 more life-years, and 824 more quality-adjusted life-years per 1000 patients compared with conventional imaging. However, these estimates are sensitive to assumptions regarding the effectiveness of earlier treatment as well as prostate-specific antigen level at imaging.

**Meaning:**

The findings suggest that assuming modest benefits of earlier detection, PSMA-PET imaging could improve the length and quality of life for patients with recurrent prostate cancer.

## Teaser

This decision analytic modeling study estimates the long-term outcomes of prostate-specific membrane antigen positron emission tomography (PSMA-PET) for staging recurrent prostate cancer.

## Introduction

Positron emission tomography (PET) imaging targeting prostate-specific membrane antigen (PSMA), a protein highly expressed on the surface of prostate cancer cells, improves the local staging and detection of occult metastatic prostate cancer compared with conventional imaging.^1,2^ Although up to one-half of patients who receive initial local treatment may develop biochemical recurrence (BCR), accurate disease localization has been elusive using conventional computed tomography and bone scan (CTBS), leading to uncertainty about the necessity and manner of salvage therapy.^3,4^ By comparison, PSMA-PET is more sensitive and specific for the detection of recurrent prostate cancer, particularly at lower prostate-specific antigen (PSA) levels indicative of smaller volumes of disease.^5,6,7^ In the phase III CONDOR multicenter study of patients with BCR, PSMA-PET detected occult disease in 59% to 66% of patients with negative conventional imaging findings.^8^ Based on improved diagnostic accuracy, several PSMA-PET radiotracers have recently been approved for the evaluation of recurrent or newly diagnosed prostate cancer and have become widely available through commercialization.^1,2,9,10^ As a result of increased detection, PSMA-PET imaging commonly leads to changes in clinical management, favoring the addition of metastasis-directed therapy (MDT) and intensified courses of systemic therapy.

As the paradigm for prostate cancer imaging changes, the long-term consequences of widely implementing PSMA-PET imaging are not known. Although regulatory approvals and practice guidelines that support the use of PSMA-PET as an alternative to conventional imaging have been based on improved diagnostic accuracy, little is known about the patient benefit of this strategy. In particular, there is a lack of clarity regarding whether the use of more sensitive forms of imaging, such as PSMA-PET, is associated with a reduction in disease progression or mortality or improved quality of life. Based on its role in prompting an array of local and systemic therapies, PSMA-PET imaging may be accompanied by risks and benefits that have not been well defined, including overtreatment of patients in whom recurrent disease may not become clinically apparent during their lifetime.^11^ In other patients, early detection of local recurrence or metastasis may allow the initiation of curative or effective therapies that prolong survival or reduce suffering from the disease. In the absence of level 1 evidence concerning the downstream effects of PSMA-PET vs conventional imaging, we aimed to estimate the long-term outcomes of these strategies using the best available evidence and to explore conditions that could optimize patient benefit from imaging.

## Methods

### Population

We applied decision analytic modeling to estimate clinical benefits and harms associated with prevailing diagnostic imaging strategies for BCR. The model considers patients diagnosed with prostate cancer who have received and recovered from initial definitive radical prostatectomy or radiation therapy and experienced BCR, defined as a persistent or rising PSA of 0.20 ng/mL after prostatectomy or PSA 2.0 ng/mL or higher following radiation therapy.^12^

This study was determined to be nonhuman participant research by the Yale University Institutional Review Board. Reporting was conducted in accordance with the 2022 Consolidated Health Economic Evaluation Reporting Standards (CHEERS) guideline.

### Decision Analytic Model

We developed a decision analytic model consisting of a decision tree and Markov model to simulate the lives of patients with BCR over a lifetime horizon (eMethods in Supplement 1). The model simulates 3 alternative diagnostic imaging strategies that patients may encounter: (1) immediate PSMA-PET without conventional imaging, (2) PSMA-PET imaging as a reflex test if CTBS findings are negative or equivocal, and (3) CTBS without PSMA-PET (ie, conventional imaging alone).

#### Decision Tree

**Figure 1.**
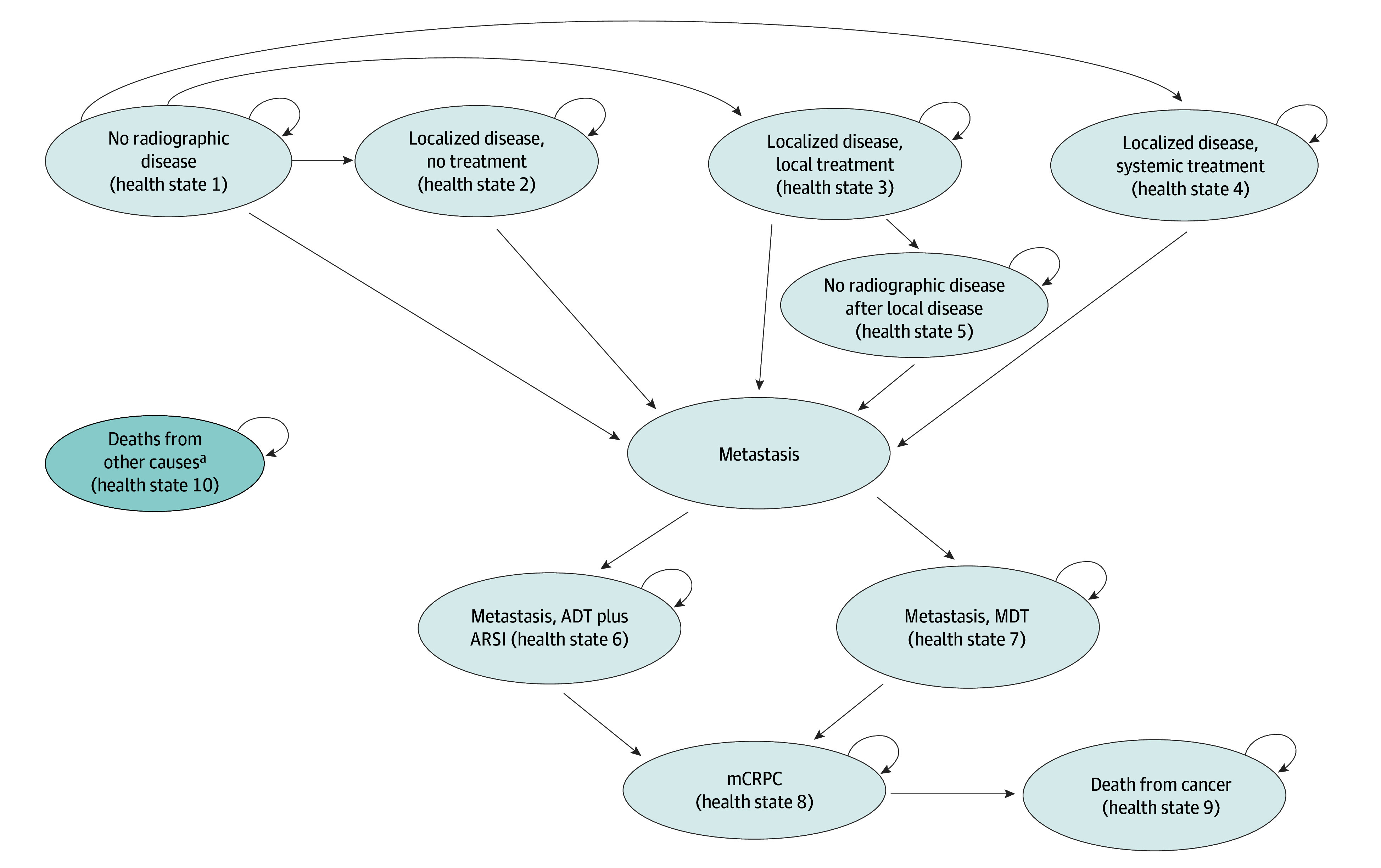
Markov Model Structure Showing Mutually Exclusive Health States That Patients May Enter of Transition Between Following the Diagnosis of Biochemical Recurrent Prostate Cancer ADT indicates androgen deprivation therapy; ARSI, androgen receptor signaling inhibitor; mCRPC, metastatic castration-resistant prostate cancer; MDT, metastasis-directed therapy. ^a^Participants may encounter background mortality due to death from noncancer causes (health state 10). Given differing diagnostic parameters, the probability of entering these health states depends on the imaging strategy. Individuals with no radiographic disease are at risk of developing either localized disease or metastasis, while those with localized disease are at risk of developing metastasis. Furthermore, among individuals with localized disease, only those with local treatment may move to health state 5 after localized disease. Once metastasis has been identified, patients will not transition back to localized disease or no radiographic disease. Individuals with metastasis are at risk of progression to mCRPC. Only those who are in the mCRPC state can die from prostate cancer. Background mortality is applied to all health states and contributes to death from any cause.

We developed a decision tree to simulate the possible diagnostic sequences of each strategy considered (Figure 1). We assumed PSMA-PET to be the criterion standard imaging modality with the highest diagnostic accuracy. Consequently, PSMA-PET (strategy 1) identifies only individuals with true-positive and true-negative disease. In strategy 2, we assumed that individuals with negative CTBS findings undergo reflex PSMA-PET, allowing for disease to be correctly identified. However, in this strategy, positive CTBS findings may be due to both true-positive and false-positive tests. In strategy 3 (CTBS alone), imaging includes true-negative and true-positive results, as well as false-negative and false-positive results.

#### Markov Model

The Markov model simulated disease progression in the population of interest, consisting of 10 mutually exclusive health states as distributed by the results of the decision tree (Figure 1). The decision tree distributes patients into 1 of the health states with initial treatment (health states 1-4 and 6-7) after initial imaging. Strategies 2 and 3 assumed that patients with false-positive and false-negative findings on imaging would receive equivalent clinical treatment as those with true-positive and true-negative findings. For strategy 3, we extended the model to include 2 additional health states—local disease false-negative findings and metastatic disease false-negative findings—and assumed that 10% of these individuals would be appropriately identified in each of the following years. We further assumed that patients with false-negative imaging findings would experience risks of disease progression that could be expressed as a hazard ratio (HR) for early (diagnosed using PSMA-PET) vs delayed (diagnosed using conventional imaging) treatment of metastasis. The base case analysis assumed lower risks of disease progression with earlier detection attributable to intervention, such as salvage local therapy for recurrent local disease, as well as earlier initiation of systemic therapy for metastatic disease.^13^ We also assumed that MDT would improve progression-free survival compared with systemic therapy, exploring these assumptions in sensitivity analyses^14,15^ (details provided in the Uncertainty Analyses section).

### Input Parameters

The decision analytic model was informed by several data sources (Table 1),^13,15,17,18,19,20,21,22,23,24,25,26,27,28^ including a retrospective study of patients who underwent PSMA-PET imaging for the evaluation of BCR at 2 large academic institutions (Yale Medicine and the Mayo Clinic) (eTable in Supplement 1). The institutional data were used to provide contemporary estimates of the imaging findings and subsequent treatment in the era following PSMA-PET approvals. Furthermore, best available evidence regarding diagnostic imaging performance, probabilities of progression between health states, utility values, and other assumptions was obtained from the published literature.^16,17,18,19,20,21,22,23,24,26,27,29^

**Table 1.** Input Parameters for Decision Model Examining Associations of PSMA-PET and Conventional Imaging Strategies With the Evaluation Outcomes of Biochemical Recurrent Prostate Cancer

Parameter	Point estimate, % (95% UI)	Distribution	Source
Assumed median age, y	66	NA	NA
Assumed discount rate, %^16^	3	NA	NA
Probability of detecting disease with PSMA-PET imaging	75.20 (71.18-78.98)	β	Yale-Mayo Clinic retrospective study^a^
Probability of no radiographic disease with PSMA-PET imaging	24.80 (21.02-28.82)	β	Yale-Mayo Clinic retrospective study^a^
Probability of localized disease among individuals with detected disease on PSMA-PET imaging	18.61 (14.79-22.83)	β	Yale-Mayo Clinic retrospective study^a^
Probability of metastatic disease among individuals with detected disease on PSMA-PET imaging	81.39 (77.17-85.21)	β	Yale-Mayo Clinic retrospective study^a^
**Transition probabilities** ^b^			
Probability of localized disease when in no radiographic disease	30.28 (19.33-42.65)	β	Schmidt-Hegemann et al,^17^ 2019
Probability of developing metastasis when in no radiographic disease	2.55 (0.49-5.90)	β	Bianchi et al,^18^ 2022
Probability of transitioning to no radiographic disease among individuals with localized disease treated with local treatment	54.55 (46-63.56)	β	Schmidt-Hegemann et al,^17^ 2019
Probability of developing metastasis when in localized disease with no treatment	4.94 (4.20-5.75)	β	Pound et al,^19^ 1999
Probability of developing metastasis when in localized disease with local treatment	2.72 (2.03-3.43)	β	Stephenson et al,^20^ 2004
Probability of developing metastasis when in localized disease with systemic treatment	5.66 (2.20-10.55)	β	Freedland et al,^21^ 2005
Probability of developing metastasis when in no radiographic disease after local disease	2.27 (1.87-2.71)	β	Stish et al,^22^ 2016
Probability of mCRPC when in metastatic disease treated with ADT plus ARSI	16.11 (14.14-18.11)	β	Davis et al,^23^ 2019
Probability of death from prostate cancer when in mCRPC^c^	34.86 (33.68-36.00)	β	Shore et al,^24^ 2021
**Other probabilities**			
Probability of receiving no treatment among individuals with no radiographic disease	54.63 (45.22-63.89)	Dirichlet	Yale-Mayo Clinic retrospective study^a^
Probability of receiving radiation among individuals with no radiographic disease	13.94 (8.14-20.94)	Dirichlet	Yale-Mayo Clinic retrospective study^a^
Probability of receiving systemic treatment among individuals with no radiographic disease	31.43 (22.79-40.23)	Dirichlet	Yale-Mayo Clinic retrospective study^a^
Probability of receiving no treatment among individuals with localized disease	33.66 (22.37-45.46)	Dirichlet	Yale-Mayo Clinic retrospective study^a^
Probability of receiving local treatment among individuals with localized disease	21.04 (11.90-31.76)	Dirichlet	Yale-Mayo Clinic retrospective study^a^
Probability of receiving systemic treatment among individuals with localized disease	45.29 (33.15-58.42)	Dirichlet	Yale-Mayo Clinic retrospective study^a^
Probability of receiving prostatectomy among individuals with localized disease treated with local treatment	2.59 (0.57-6.05)	Dirichlet	Yale-Mayo Clinic retrospective study^a^
Probability of receiving radiation among individuals with localized disease treated with local treatment	85.93 (78.63-91.46)	Dirichlet	Yale-Mayo Clinic retrospective study^a^
Probability of receiving cryotherapy among individuals with localized disease treated with local treatment	11.48 (6.40-17.94)	Dirichlet	Yale-Mayo Clinic retrospective study^a^
Probability of receiving systemic treatment among individuals with metastatic disease	79.62 (74.72-84.26)	β	Yale-Mayo Clinic retrospective study^a^
Probability of receiving metastasis-directed treatment among individuals with metastatic disease	20.38 (15.75-25.28)	β	Yale-Mayo Clinic retrospective study^a^
**Hazard ratios**			
For mCRPC from metastatic disease treated with metastasis-directed vs ADT plus ARSI therapy	0.25 (0.12-0.55)	Log-normal	Tang et al,^15^ 2023
For progression with early (diagnosed with PSMA-PET) vs delayed (conventional imaging) treatment	0.56 (0.49-0.92)	Log-normal	Meijer et al,^13^ 2022
For shorter time to disease progression among individuals with false-negative findings on conventional imaging (95% CI)	1.79 (1.09-2.05)	Log-normal	Meijer et al,^13^ 2022
**Test accuracy**			
Sensitivity of CTBS	38 (24-52)	β	Hofman et al,^25^ 2020
Specificity of CTBS	91 (85-97)	β	Hofman et al,^25^ 2020
**Utility values**			
No radiographic disease	0.90 (0.84-0.96)	β	Torvinen et al,^26^ 2013
No radiographic disease after localized disease	0.90 (0.84-0.96)	β	Torvinen et al,^26^ 2013
Localized disease with no treatment	0.90 (0.84-0.96)	β	Torvinen et al,^26^ 2013
Localized disease treated with local treatment	0.83 (0.81-0.88)^d^	β	Stewart et al,^27^ 2005
Localized disease treated with systemic treatment	0.83 (0.78-0.98)	β	Stewart et al,^27^ 2005
Metastatic disease treated with ADT plus ARSI	0.80 (0.76-0.84)	β	Chi et al,^28^ 2018
Metastatic disease treated with metastasis-directed treatment	0.72 (0.69-0.75)	β	Stewart et al,^27^ 2005
mCRPC	0.63 (0.58-0.67)	β	Schmidt-Hegemann et al,^17^ 2019

Abbreviations: ADT, androgen deprivation therapy; ARSI, androgen receptor signaling inhibitor; CTBS, computed tomography and bone scan; mCRPC, metastatic castration-resistant prostate cancer; NA, not applicable; PET, positron emission tomography; PSMA, prostate-specific membrane antigen; UI, uncertainty interval.

^a^ Details provided in the eTable in Supplement 1.

^b^ All probabilities were converted to 1-year probabilities.

^c^ Based on median overall survival in months after initiating first-line therapy for mCRPC.

^d^ Point estimate incorporating short-term toxicity due to salvage local therapies.

### Uncertainty Analyses

We propagated uncertainty from model input parameters to the outcomes of the model using a probabilistic analysis, first informing all input parameters with appropriate probability distributions and then conducting a Monte Carlo simulation with 10 000 iterations. We further performed sensitivity and scenario analyses to examine the impact of specific assumptions and input parameters associated with our results. The base case analysis considered patients with a median PSA level of 1.8 ng/mL at imaging based on the observed values in a retrospective analysis at our institutions. As PSMA-PET findings vary by PSA level, we performed scenario analyses with different PSA levels at the time of imaging reflecting the distribution of PSA values seen in our study and reported in the literature,^30^ specifying the following a priori categories: 0-1.99 ng/mL; 2.00-4.99 ng/mL; and ≥5.00 ng/mL. In these exploratory analyses, we varied the probability of specific image findings and management but maintained all other model parameters. Next, we explored assumptions regarding the difference in disease progression that would result from delayed treatment due to false-negative imaging results. For this purpose, we applied a base case HR of 1.79 (95% CI, 1.09-2.05) for accelerated disease progression with delayed (strategy 3) vs earlier detection.^13^ Given lower-quality evidence for this estimate, we assessed the impact of this assumption on our results. We explored clinical outcome estimates that could arise if early detection would be harmful (ie, an HR for progression among patients with false-negative results <1.00), as well as when increasing the beneficial outcomes of the early diagnosis beyond the base case estimate. To explore the outcomes of MDT, we applied a base case HR estimate of 0.25 (95% CI, 0.12-0.55) for progression to metastatic castration-resistant prostate cancer from metastatic disease with the addition of MDT based on estimates from a phase II randomized clinical trial and further varied these estimates in the range of 0.125 to 1.000, assuming that the addition of MDT would not worsen survival.^15^ In addition, we explored assumptions regarding the diagnostic accuracy of the imaging strategies, varying the diagnostic sensitivity and specificity of CTBS between 0.15 to 0.65 and 0.40 to 1.00, respectively.^25^

### Statistical Analysis

The decision analytic model was used to simulate and compare the outcomes of the 3 imaging strategies.^31^ The clinical outcomes considered included local recurrences detected, metastases detected, proportion of patients receiving various treatments among those with no radiographic disease, localized disease and metastatic disease, deaths from prostate cancer, life-years, and quality-adjusted life-years (QALYs) for each of the strategies considered. All analyses were conducted using R, version 4.1.3 software (R Foundation for Statistical Computing) between April 1, 2023, and May 1, 2024.

## Results

The projected outcomes of interest according to the 3 imaging strategies are presented in Table 2. Per 1000 patients with BCR (assumed median age, 66 years), upfront PSMA-PET alone (strategy 1) was estimated to detect 611 patients (95% uncertainty interval [UI], 565-656 patients) with metastatic disease, 140 patients (95% UI, 109-177 patients) with localized disease, and 249 patients (95% UI, 212-290 patients) with no radiographic disease. Imaging with PSMA-PET as a reflex test if CTBS findings are negative or equivocal (strategy 2) was estimated to detect 630 patients (95% UI, 585-675 patients) with metastatic disease, 144 patients (95% UI, 112-181 patients) with localized disease, and 226 patients (95% UI, 188-267 patients) with no radiographic disease per 1000 patients. Conventional imaging alone (strategy 3) was estimated to detect 297 patients (95% UI, 202-410 patients) with metastatic disease, 10 patients (95% UI, 2-25 patients) with localized disease, and 692 patients (95% UI, 576-792 patients) with no radiographic disease per 1000 patients. As a result of increased detection, PSMA-PET strategies 1 and 2 were estimated to result in more patients initiating systemic therapy and MDT compared with conventional imaging alone.

**Table 2.** Disease Detection and Treatment Initiation for Each Imaging Strategy

Variable	Strategy, No. of patients per 1000 (95% UI)		
	PSMA-PET alone	PSMA-PET if conventional imaging findings negative	Conventional imaging alone
**Disease detection**			
No radiographic disease	249 (212-290)	226 (188-267)	692 (576-792)
Localized disease	140 (109-177)	144 (112-181)	10 (2-25)
Metastatic disease	611 (565-656)	630 (586-675)	297 (202-410)
**Treatment initiation**			
No radiographic disease			
No treatment	136 (106-170)	124 (95-156)	378 (287-463)
Local treatment	34 (19-53)	31 (18-48)	95 (54-143)
Systemic therapy	79 (54-105)	72 (49-96)	219 (151-292)
Localized disease			
No treatment	47 (29-69)	48 (30-71)	3 (1-10)
Local treatment	29 (16-47)	30 (17-48)	2 (0-6)
Systemic therapy	63 (41-87)	65 (42-90)	5 (1-11)
Metastatic disease			
Systemic therapy	486 (438-531)	501 (451-546)	236 (157-331)
Metastasis-directed treatment	125 (97-159)	129 (99-164)	61 (38-90)

Abbreviations: PET, positron emission tomography; PSMA, prostate-specific membrane antigen; UI, uncertainty interval.

Compared with conventional imaging alone, PSMA-PET imaging was estimated to increase the number of life-years by 988 (95% UI, 821-1146 life-years) or 824 QALYs (95% UI, 698-885 QALYs) and lead to 75 (95% UI, 66-81) fewer deaths from prostate cancer per 1000 patients with BCR (Table 3). Similarly, PSMA-PET imaging as a reflex test if CTBS findings are negative or equivocal was estimated to result in an expected increase in life expectancy by 854 life-years (95% UI, 683-1015 life-years) or 693 QALYs (95% UI, 561-763 QALYs) and a decrease in prostate cancer mortality by 67 deaths (95% UI, 58-73 deaths) compared with conventional imagining.

**Table 3.** Estimated Life-Years, QALYs, and Deaths From Prostate Cancer per 1000 Patients With Biochemical Recurrent Prostate Cancer Undergoing Each Imaging Strategy^a^

Variable	Strategy, No. (95% UI)		
	PSMA-PET alone	PSMA-PET if conventional imaging findings negative^b^	Conventional imaging alone^b^
**All participants**			
Life-years	10 987 (10 437-11 528)	10 853 (10 305-11 390)	9999 (9291-10 707)
QALYs	8741 (8165-9 289)	8609 (8043-9152)	7917 (7280-8591)
Deaths from prostate cancer	512 (472-552)	520 (480-559)	587 (538-632)
**PSA 0-1.99 ng/mL**			
Life-years	12 321 (11 610-13 028)	12 134 (11 408-12 845)	11 400 (10 598-12 184)
QALYs	9939 (9216-10 680)	9754 (9015-10 491)	9155 (8333-9953)
Deaths from prostate cancer	427 (374-478)	438 (384-490)	498 (440-552)
**PSA 2.00-4.99 ng/mL**			
Life-years	10 306 (9379-11 280)	10 229 (9311-11 175)	9252 (8221-10 324)
QALYs	8025 (7200-8940)	7949 (7149-8876)	7179 (6240-8182)
Deaths from prostate cancer	549 (488-611)	554 (493-613)	630 (566-693)
**PSA ≥5.00 ng/mL**			
Life-years	9243 (8551-9925)	9204 (8522-9896)	8208 (7476-8980)
QALYs	7169 (6546-7822)	7132 (6524-7793)	6313 (5642-7027)
Deaths from prostate cancer	621 (577-664)	623 (581-666)	697 (650-740)

Abbreviations: PET, positron emission tomography; PSA, prostate-specific antigen; PSMA, prostate-specific membrane antigen; QALY, quality adjusted life-year; UI, uncertainty interval.

^a^ Results further stratified by PSA level at the time of imaging.

^b^ Conventional imaging consists of computerized tomography and bone scan.

### PSA-Stratified Analyses

Analysis by PSA level suggested benefit of PSMA-PET imaging in all PSA strata examined (Table 3). However, the greatest relative benefit to PSMA-PET compared with conventional imaging was observed at the highest PSA distribution (≥5.00 ng/mL). In this subset, PSMA-PET vs conventional imaging was estimated to lead to an increase of 1035 life-years (95% UI, 945-1075 life-years) and 856 QALYs (95% UI, 795-904 QALYs) and to 76 fewer deaths (95% UI, 73-76 deaths) from prostate cancer per 1000 patients. Among patients with PSA levels 0 to 1.99 ng/mL, use of upfront PSMA-PET vs conventional imaging was estimated to result in an increase of 921 life-years (95% UI, 845-1013 life-years) and 784 QALYs (95% UI, 727-883 QALYs) and 71 fewer deaths (95% UI, 66-74 deaths) from prostate cancer.

### Scenario Analyses

**Figure 2.**
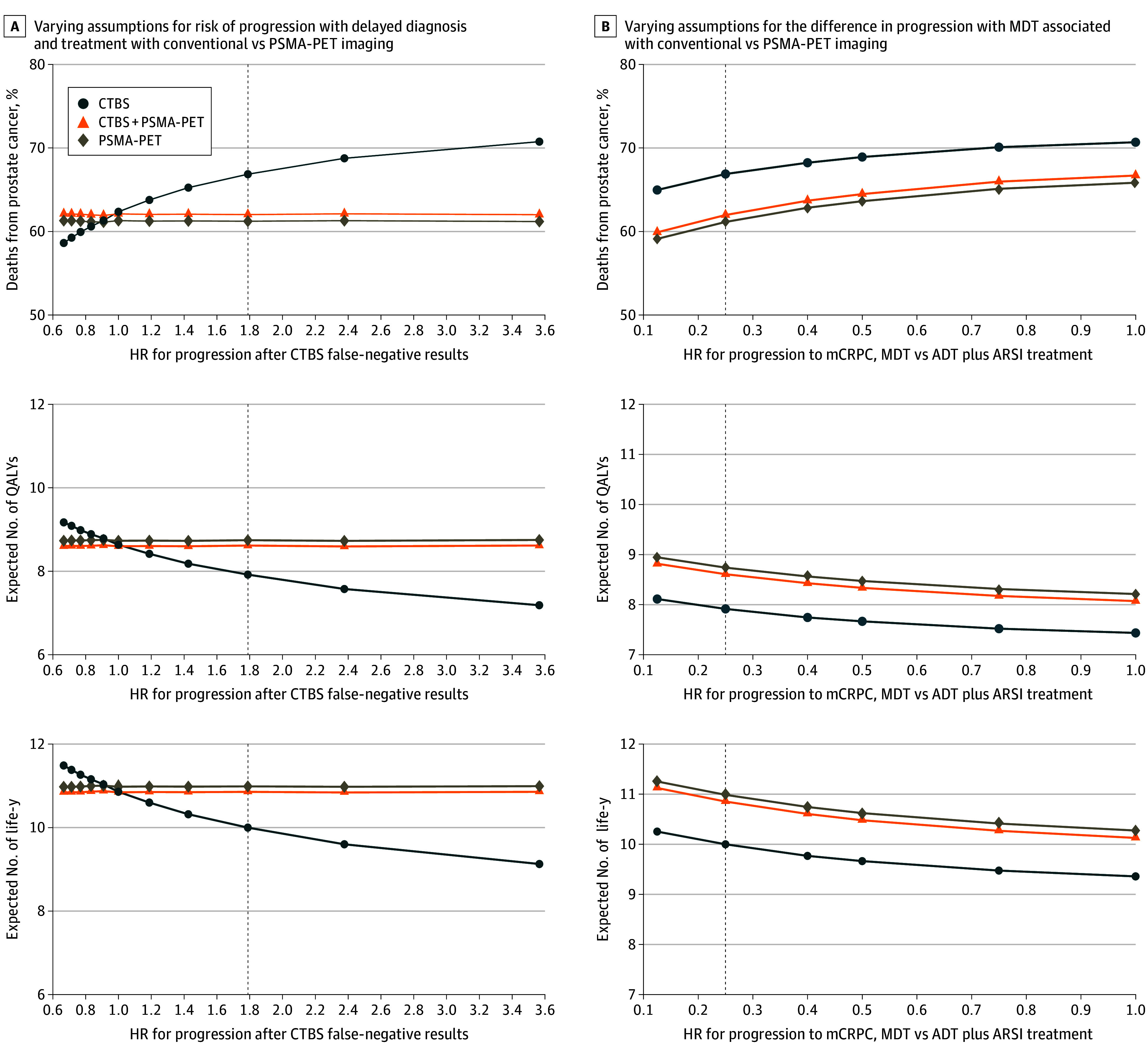
Results of Sensitivity Analyses A, Dashed line indicates base case hazard ratio (HR) of 1.79. B, Dashed line indicates base case HR of 0.25. Conventional imaging includes computed tomography and bone scan (CTBS). ADT indicates androgen deprivation therapy; ARSI, androgen receptor signaling inhibitor; mCRPC, metastatic castration-resistant prostate cancer; MDT, metastasis-directed therapy; PET, positron emission tomography; PSMA, prostate-specific membrane antigen; QALY, quality-adjusted life-year.

Scenario analyses revealed that if delayed diagnosis with conventional imaging would increase the HR for progression by 19%, PSMA-PET may result in 386 more life-years and 317 more QALYs compared with conventional imaging (Figure 2A). When varying the effect estimate to assume no benefit of MDT, the PSMA-PET strategies were associated with more life-years, more QALYs, and fewer deaths from prostate cancer (Figure 2B). Finally, even when assuming higher sensitivity or specificity of CTBS, PSMA-PET strategies were still estimated to improve long-term outcomes (eFigure in Supplement 1).

## Discussion

To address the evidence gap regarding long-term outcomes of integrating molecular imaging into the evaluation paradigm for recurrent prostate cancer, we developed a decision analytic model to estimate clinical outcomes associated with PSMA-PET vs conventional imaging strategies. Our results suggest that use of PSMA-PET alone or as a reflex test following negative findings with conventional imaging is expected to detect more than twice the number of patients with metastatic disease and lead to the initiation of systemic therapy in more than 60% of patients imaged. The PSMA-PET imaging strategies are estimated to result in nearly 1 additional life-year and 0.8 QALYs per patient imaged. Although the distribution of imaging findings varied by PSA level, PSMA-PET strategies yielded more life-years and QALYs and fewer deaths from prostate cancer across all strata explored. Importantly, the estimates from this model are sensitive to the expected benefit of initiating therapy for recurrent prostate cancer earlier in the disease course but suggest that even modest (eg, 20%) reductions in the HR for disease progression with delayed vs earlier treatment may support the benefit of PSMA-PET detection. In light of rapid clinical uptake, we show numerous clinically relevant findings to anticipate the effects of new developments in the imaging landscape for prostate cancer.

The results of this model underscore that the management of prostate cancer may change as a result of the widespread uptake of PSMA-PET imaging. Drawn from contemporary findings obtained from imaging in the era following approval of PSMA-PET, our results suggest that the majority of patients imaged for BCR would have radiographic disease and initiate systemic therapy, including MDT, in more than twice the proportion of patients undergoing conventional imaging. These findings not only agree with results of prospective studies that defined the diagnostic yield of PSMA-PET imaging but also highlight the potential for PSMA-PET imaging to facilitate the earlier and potentially indefinite use of systemic therapy in patients identified to have otherwise occult metastatic disease by conventional imaging.^32^

Projected gains in life-years and QALYs and reductions in deaths from prostate cancer were driven by earlier initiation of salvage therapies, including both curative local therapies and metastasis-directed radiation therapy and systemic agents. These estimates are consistent with a prior cost-effectiveness study published from the Australian health care perspective and built on strategies for considering long-term effectiveness of diagnostic imaging.^33^ In the Australian model, the investigators applied a single HR estimate for the risk of death associated with delayed diagnosis.^33,34^ However, to explore the possibility that the outcomes of early treatment for lower-volume, PSMA-PET–evident disease may not improve, we conducted scenario analyses to define the boundaries at which earlier diagnosis and treatment could improve long-term outcomes. As expected, gains in life-years and QALYs or reductions in death from prostate cancer associated with PSMA-PET imaging decreased with lessened benefit of earlier treatment.

While the diagnostic imaging yield of PSMA-PET is known to vary by PSA level at the time of imaging, our findings suggest clinical benefit across the range of values encountered in real-world practice. Owing to a lower prevalence of PSMA-PET–evident localized or distant metastatic disease at lower PSA values, the base case analysis estimated a lower absolute benefit to imaging; however, improvements in outcomes were still anticipated in patients with PSA less than 2.00 ng/mL. These findings correspond to studies that have shown diagnostic yields with PSMA-PET imaging even at very-low (<0.20 ng/mL) PSA levels, including early detection of oligometastatic disease.^35^ Greater projected benefit among patients with higher PSA levels may be driven by wider diagnostic gaps in detection of radiographic disease between PSMA-PET and conventional imaging and associated differences in treatment initiation.

To our knowledge, this study is the first to address outcomes of PSMA-PET in the setting of recurrence in the US population. Informed by observed patterns of imaging and treatment initiation in the real-world setting, the results from this model highlight the cascade of treatments that may be initiated following more sensitive forms of molecular imaging. In the context of cancer imaging, relevant outcomes, such as overall survival or quality of life, are frequently not available due to a limited follow-up period. However, our findings highlight the need to consider longer evaluation horizons, as well as methodological strategies to inform preliminary boundaries at which imaging agents may yield improved patient outcomes.

### Limitations

This study has several limitations. The model integrates assumptions that treatment delivered as a result of earlier detection is associated with improved locoregional control and progression-free survival, even in the setting of metastatic disease. However, the randomized clinical trials used to inform this base estimate were conducted without PSMA-PET–based imaging definitions of metastasis and commonly used androgen deprivation therapy as monotherapy rather than intensified multiagent regimens now recommended in the first-line setting.^34^ To account for uncertainty in these estimates, we conducted probabilistic and scenario analyses to explore the dependence of the model output on assumptions of treatment effectiveness, diagnostic accuracy of conventional imaging, and PSA levels at the time of imaging. The inputs from our retrospective study are drawn primarily from patients with lower PSA distributions, a representative sample of patients undergoing PSMA-PET but a subset in whom conventional imaging has poor diagnostic yield. Thus, these estimates cannot necessarily be extrapolated to patients with very high PSA levels (eg, ≥20 ng/mL) in whom detection of metastasis is likely with CTBS and may not meaningfully alter management strategies or outcomes. Although we performed scenario analyses by PSA level at the time of imaging, we did not directly account for differences by type of local therapy or clinical reasoning influencing the decision to undergo imaging. Differences in definitions of BCR between patients treated with radiation vs prostatectomy may introduce biases between these groups that should be accounted for in subsequent studies. Furthermore, as the Markov model structure introduces limitations related to patient and time heterogeneity, future studies should also incorporate patient heterogeneity, as well as time-dependent risks and outcomes. In addition, we assumed that PSMA-PET imaging would accurately characterize disease status and did not account for the possibility of equivocal findings or variations in reader interpretation. Finally, this model did not consider economic costs and was limited to a single imaging event without the possibility of sequential imaging.

## Conclusions

This decision analytic modeling study projected that upfront PSMA-PET for the imaging of BCR prostate cancer is expected to lead to more patient life-years and QALYs and fewer deaths from prostate cancer. Clinical benefits were expected across PSA values. The benefits associated with earlier detection and initiation of salvage therapies were sensitive to assumptions regarding the effectiveness of early treatment.

## Notes

Supplement 1. **eTable.** Results of Institutional Analysis Informing the Distribution of PSMA-PET Findings for Evaluation of Biochemical Recurrent Prostate Cancer and Subsequent Treatment Initiation **eFigure.** Sensitivity Analysis Varying the Diagnostic Sensitivity (panel A) and Specificity (panel B) of CT and Bone Scan Imaging
eMethods.

Supplement 2.
Data Sharing Statement
