# Supplementary material for: Long-Term Outcomes of Prostate-Specific Membrane Antigen–PET Imaging of Recurrent Prostate Cancer
Source: JAMA Netw Open. 2024 Oct 23;7(10):e2440591. doi: 10.1001/jamanetworkopen.2024.40591 (PMC11581571; doi:10.1001/jamanetworkopen.2024.40591)
Supplement: Supplement 1. — eTable. Results of Institutional Analysis Informing the Distribution of PSMA-PET Findings for Evaluation of Biochemical Recurrent Prostate Cancer and Subsequent Treatment Initiation eFigure. Sensitivity Analysis Varying the Diagnostic Sensitivity (panel A) and Specificity (panel B) of CT and Bone Scan Imaging eMethods. [file jamanetwopen-e2440591-s001.pdf]

# Supplemental Online Content

Kunst N, Long JB, Westvold S, et al. Long-term outcomes of prostate-specific membrane antigen–PET imaging of recurrent prostate cancer. *JAMA Netw Open*. 2024;7(10):e2440591. doi:10.1001/jamanetworkopen.2024.40591

**eTable.** Results of Institutional Analysis Informing the Distribution of PSMA-PET Findings for Evaluation of Biochemical Recurrent Prostate Cancer and Subsequent Treatment Initiation

**eFigure.** Sensitivity Analysis Varying the Diagnostic Sensitivity (panel A) and Specificity (panel B) of CT and Bone Scan Imaging

**eMethods.**

This supplemental material has been provided by the authors to give readers additional information about their work.

© 2024 Kunst N et al. *JAMA Network Open.*

**eTable.** Results of Institutional Analysis Informing the Distribution of PSMA-PET Findings for Evaluation of Biochemical Recurrent Prostate Cancer and Subsequent Treatment Initiation

Panel A describes imaging findings and subsequent treatment across all PSA values at the time of PSMA-PET imaging. In Panel B, imaging results and subsequent management are stratified by PSA categories.

A.

| | | | | | | | | | | |
|---|---|---|---|---|---|---|---|---|---|---|
| | **Any N=441** | **%** | **Local N=86** | **%** | **Nodal N=61** | **%** | **Metastatic N=294** | **%** | **None N=142** | **%** |
| **No Treatment** | 68 | 15% | 29 | 34% | 17 | 28% | 22 | 7% | 79 | 56% |
| **Local** | 29 | 7% | 19 | 22% | 3 | 5% | 7 | 2% | 18 | 13% |
| **Systemic Therapy** | 258 | 59% | 34 | 40% | 35 | 57% | 189 | 64% | 36 | 25% |
| **Metastasis Directed Therapy** | 86 | 20% | 4 | 5% | 6 | 10% | 76 | 26% | 9 | 6% |

B.

| | | | | | | |
|---|---|---|---|---|---|---|
| **PSA 0-1.99 ng/mL** | | | | | | |
| | **Any** | **None** | | **Local** | **Nodal** | **Metastatic** |
| **No Treatment** | 20% | 57% | | 30% | 34% | 11% |
| **Local** | 8% | 16% | | 20% | 6% | 3% |
| **Systemic Tx** | 49% | 22% | | 45% | 54% | 49% |
| **Metastasis Directed Therapy** | 23% | 6% | | 5% | 6% | 37% |
| | | | | | | |
| **PSA 2-4.99 ng/mL** | | | | | | |
| | **Any** | **None** | | **Local** | **Nodal** | **Metastatic** |
| **No Treatment** | 13% | 6% | | 35% | 20% | 6% |
| **Local** | 7% | 1% | | 30% | 0% | 1% |
| **Systemic Tx** | 54% | 63% | | 25% | 50% | 63% |
| **Metastasis Directed Therapy** | 26% | 30% | | 10% | 30% | 30% |
| | | | | | | |
| **PSA≥5.00 ng/mL** | **Any** | **None** | | **Local** | **Nodal** | **Metastatic** |
| **No Treatment** | 11% | 40% | | 41% | 14% | 5% |
| **Local** | 4% | 0% | | 18% | 0% | 2% |
| **Systemic Tx** | 74% | 60% | | 41% | 79% | 79% |
| **Metastasis Directed Therapy** | 11% | 0% | | 0% | 7% | 14% |

© 2024 Kunst N et al. *JAMA Network Open.*

**eFigure.** Sensitivity Analysis Varying the Diagnostic Sensitivity (panel A) and Specificity (panel B) of CT and Bone Scan Imaging

**A**

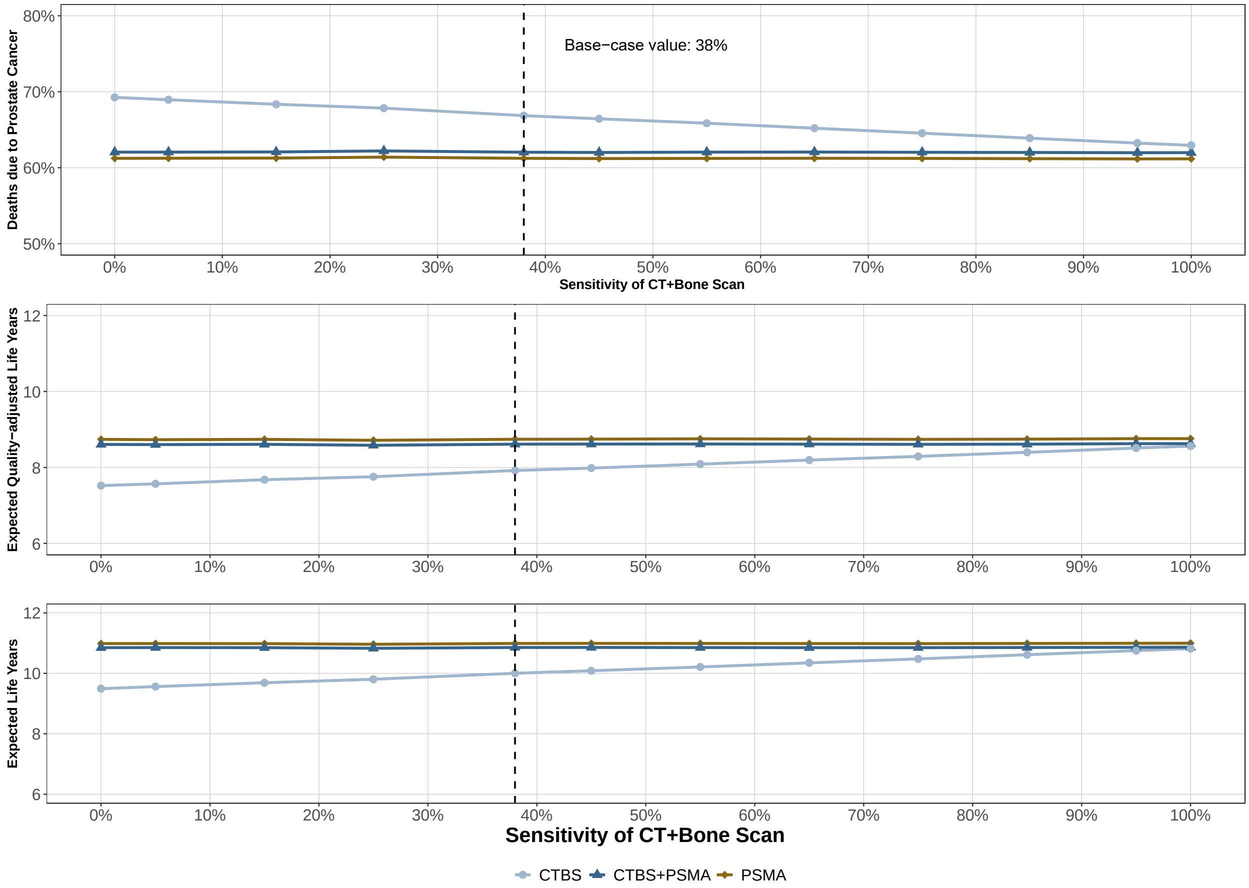


© 2024 Kunst N et al. *JAMA Network Open.*

**B**

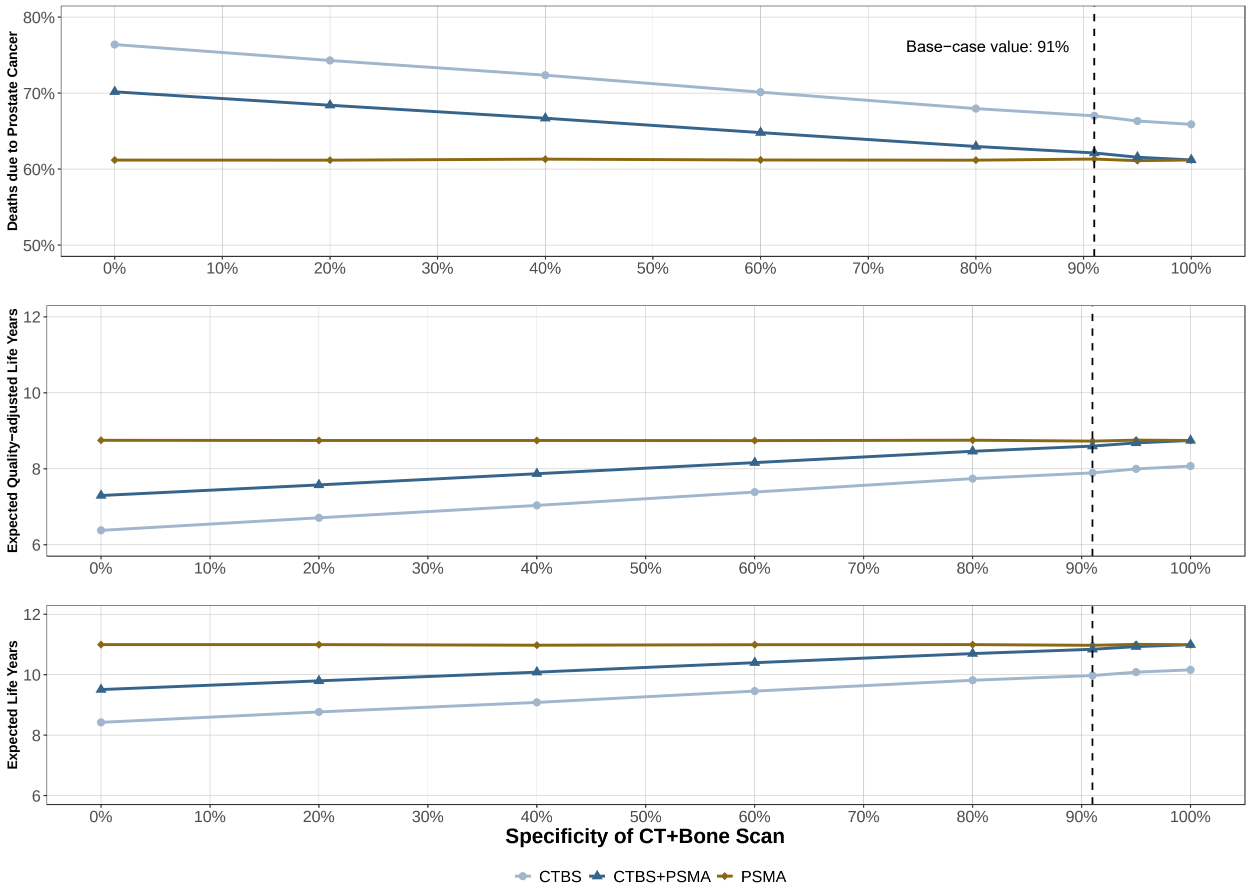


© 2024 Kunst N et al. *JAMA Network Open.*

**eMethods**

This analysis uses a decision analytic model to estimate the potential long-term consequences of implementing PSMA-PET based detection strategies for recurrent prostate cancer in comparison with the conventional approaches of cross sectional imaging and bone scintigraphy. A decision-analytic model is a mathematical framework that simplifies clinical reality by defining a number of relationships between a disease's health states and diagnostic and treatment pathways.[35 36 37] Decision-analytic models make use of a wide range of evidence and predict a set of outcomes of interest. The theoretical foundations of decision-analytic modeling are grounded in statistical decision theory and are also closely associated with Bayesian statistics. [38 39 40] In our study, we developed a Markov model to simulate life expectancy expressed in average life years (LYs), quality-adjusted life expectancy expressed in average quality-adjusted life years (QALYs) and other diagnostic and clinical outcomes of interest. This model allows us to simulate a patient cohort's journey through different health states over time allowing transition between health states (e.g., disease-free, disease progression, and death) at defined transition probabilities.[27] Each state is associated with a specific risk to transition to other health states (e.g., progressed disease), risk of mortality (due to the disease and other causes), and quality of life. Background mortality, which represents the risk of death from causes other than the simulated disease, is also incorporated into the Markov model. This is achieved by including a transition probability from each health state to the 'death' state that is estimated based on the age-specific and general population mortality rates. By accounting for background mortality, the model provides a more holistic estimate of life expectancy and health outcomes, ensuring that the effects of PSMA PET is accurately assessed within the context of overall patient mortality. The model outputs for expected life years, QALYs and other diagnostic and clinical outcomes under different scenarios, are derived by simulating these transitions over a lifetime horizon.

© 2024 Kunst N et al. *JAMA Network Open.*
