# Supplementary material for: Long-Term Outcomes of Prostate-Specific Membrane Antigen–PET Imaging of Recurrent Prostate Cancer
Source: JAMA Netw Open. 2024 Oct 23;7(10):e2440591. doi: 10.1001/jamanetworkopen.2024.40591 (PMC11581571; doi:10.1001/jamanetworkopen.2024.40591)
Supplement: Supplement 2. — Data Sharing Statement [file jamanetwopen-e2440591-s002.pdf]

Data Sharing Statement

Kunst. Long-Term Outcomes of Prostate-Specific Membrane Antigen–PET Imaging of Recurrent Prostate Cancer. *JAMA Netw Open*. Published October 23, 2024. doi:10.1001/jamanetworkopen.2024.40591

Data
Data available: Yes
Data types: Data (not involving human participants)
How to access data: Data input parameters will be made available. No patient level data was collected for this simulation study.
When available: With publication

Supporting Documents
Document types: Other (please specify)
Additional Information: Data input parameters will be made available. No patient level data was collected for this simulation study.
How to access documents: Appendix
When available: With publication

Additional Information
Who can access the data: Researchers
Types of analyses: Modeling
Mechanisms of data availability: After approval
